# Sequence analysis of the cytochrome c oxidase subunit 1 gene of *Sarcoptes scabiei* isolated from goats and rabbits in East Java, Indonesia

**DOI:** 10.14202/vetworld.2019.959-964

**Published:** 2019-07-05

**Authors:** Nunuk Dyah Retno Lastuti, Ali Rohman, Didik Handiyatno, Dony Chrismanto, Kurnia Desiandura

**Affiliations:** 1 Department of Veterinary Parasitology, Faculty of Veterinary Medicine, Universitas Airlangga, Surabaya, Indonesia; 2 Department of Chemistry, Faculty of Science and Technology, Universitas Airlangga, Surabaya, Indonesia; 3 Department of Veterinary Microbiology, Faculty of Veterinary Medicine, Universitas Airlangga, Surabaya, Indonesia; 4 Study Program of Animal Health, Faculty of Vocational, Universitas Airlangga, Surabaya, Indonesia; 5 Master Program Student, Faculty of Veterinary Medicine, Universitas Airlangga, Surabaya, Indonesia

**Keywords:** cytochrome c oxidase-1, East Java, goat, Indonesia, rabbit, *Sarcoptes scabiei*

## Abstract

**Aim:**

This study aimed to sequence the Cytochrome c oxidase (*COX-1*) gene sequence from mitochondrial DNA of *Sarcoptes scabiei* isolated from Lamongan goats and Mojokerto rabbits, align it with DNA isolated from Zi’gong rabbit (GenBank accession No. EU256389.1), and produce a phylogenetic analysis of *S. scabiei* *COX-1* gene.

**Materials and Methods:**

*S. scabiei* mites were obtained from goats and rabbits, and DNA was extracted using QIAamp DNA Mini Kit. The forward and reverse primer sequences were designed based on the DNA sequence of an *S. scabiei* *COX-1* gene isolated from the Zi’gong rabbit (5’-TCTTAGGGGCTGGATTTAGTATG-3’ and 5’-AGTTCCTCTACCAGTTCCAC-3’, respectively). To confirm sequencing output, the sequence resulting from the reverse primer was inverted and aligned to the sequence from the forward primer using Clone Manager Professional Version 9 for Windows (Scientific & Educational Software; http://www.scied.com). This alignment was subsequently used to build a phylogenetic tree, using the Neighbor-Joining method, in the MEGA6 program (https://www.megasoftware.net/).

**Results:**

Polymerase chain reaction (PCR) products from *S. scabiei* isolates from Lamongan goats and Mojokerto rabbits produced bands of around 290 bp with 2% agarose gel electrophoresis. Comparing the DNA sequences of the *S. scabiei COX-1* gene with those isolated from Lamongan goats and Mojokerto rabbits showed 99% homology.

**Conclusion:**

PCR products of the *S. scabiei* *COX-1* gene isolated from Lamongan goats and Mojokerto rabbits were around 290 bp long. The sequences had more than 99% homology. The sequences of the *COX-1* gene of *S. scabiei* from Lamongan goats and Mojokerto rabbits were relatively close to the sequence of the gene in *S. scabiei* obtained from various hosts according to National Center for Biotechnology Information data.

## Introduction

Scabies an infectious skin disease produced by the *Sarcoptes scabiei* mite affects both humans and animals. Outbreaks in domestic animals and wild mammals cause morbidity, mortality, and huge economic losses [1,2]. Scabies is very contagious and produces pruritic dermatitis, alopecia, hyperkeratosis, and crust formation [3,4]. Histopathological changes, manifest as lesions, parakeratosis, acanthosis, congestion, inflammation, and cell degeneration [5]. Scabies causes global health problems as an infectious disease which appears and re-appears [6]. Scabies outbreaks have been reported in industrialized countries, and problems caused by the disease in developing countries are increasing [7]. There are problems due to drug resistance, drug residue, and toxicity from extensive use of acaricides, especially in developing countries [8]. The *S. scabiei* mite has a range of hosts. Although there are no differences in the *S. scabiei* mite’s morphology found on different animal species, they generally show low cross-infectivity [9]. Experiments show no transmission of scabies from dog to rat, marmot, pig, cow, cat, sheep, or goat [10,11]. Research was also conducted by Harumal *et al*. [12] using the *S. scabiei antigen* 1 from cloning *S. scabiei* var. *hominis* in rabbits. The result was less protective after challenge, but the rabbits did not develop crusting.

There are ongoing genetic studies of *S. scabiei*. Analysis of a 450bp nucleotide ribosomal marker on the second internal transcribed spacer (ITS2) of *S. scabiei* such as dog, cow, fox, wombat, and dromedary found no differences [13]. There is still limited, research on developing molecular diagnostic tools for scabies because it is difficult to isolate the *Sarcoptes* mite. Microscopic examination of skin scrapings shows a positive result in only about 30-50% of scabies cases with crusta papula [8]. There is no immunodiagnostic test or commercial subunit vaccine available for scabies. Some research has focused on producing a recombinant protein from *S. scabiei*, aimed investigating the host’s immune response, to develop a subunits vaccine [1,14]. Based on field observations, the number of scabies cases in goats and rabbits in Indonesia increasing; however, there have been no case reports because the condition has been treated with ivermectin and acaricide.

The Cytochrome c oxidase (*COX-1*) gene is often used by researchers for genetic characterization of *S. scabiei* from animals and humans. The *COX-1* fragments are often used for DNA barcoding to differentiate between species. These fragments rarely experience amino acid substitutions, but silent mutations often occur. The *COX-1* fragment is useful for reconstructing phylogenetic diversity in evolutionary branches below the species level. Mitochondria undergo rapid evolution, but there are parts of it, such as the *COX-1* fragment that experience low evolution, rates and can, thus, be used as genetic characters [9,15,16].

This study aimed to sequence the *COX-1* gene sequence from mitochondrial DNA of *S. scabiei* isolated from Lamongan goats and Mojokerto rabbits, align it with DNA isolated from Zi’gong rabbit (GenBank accession No. EU256389.1), and produce a phylogenetic analysis of *S. scabiei* *COX-1* gene.

## Materials and Methods

### Ethical approval

The research complied with the ethics guidelines for using experimental animals and was approved by the Ethics Commission of the Faculty of Veterinary Medicine, Universitas Airlangga, Indonesia, No: 630-KE.

### Isolation of *S. scabiei* mites

*S. scabiei* mite samples were collected from ten rabbits and six goats infected with scabies with severe clinical symptoms, including skin thickening, crust formation, and hair loss around their eyes, ears, mouths, and legs. The goats were from husbandries at Lamongan and the rabbits from Mojokerto, East Java, Indonesia. About 250 mg of crusted skin was scraped with a sterile scalpel from the goats’ and rabbits’ ears, washed in Petri dishes with phosphate-buffered saline (PBS), and filtered to remove skin debris. The washing process was repeated 3 times. As a final washing step, the crusted skin was transferred to a 1.5 ml microcentrifuge tube, washed with 1 ml PBS by vortexing, and centrifuged at 3.000 revolutions/min (rpm) for 10 min. The filtrate was discarded, and the clean, crusted skin was stored at −20°C until used [2,4].

### Extraction of mitochondrial DNA

DNA extraction was performed using QIAamp DNA Mini Kits (Qiagen, Hilden, Germany), following the manufacturer’s protocol as follows: 20 µl of Qiagen protease (20 mg/ml) was added to a microcentrifuge tube with a 200 µl suspension of *S. scabiei*; and 180 µl of buffer tissue lysis (ATL buffer) was then added and the tube was centrifuged at 8,000 G for 3 min, vortexed for 15 s, and incubated at 56°C for 24 h. Then, 200 µl of buffer lysis (AL buffer) was added to the tube and vortexed for 15 s; 200 µl of 96% ethanol was added to the tube and vortexed for 15 s. The materials were removed, placed into a column tube, and centrifuged at 8,000 G for 1 min to pellet the DNA. Next, 500 ml of washing buffer 1 (AW1 buffer) was added to the pellet and centrifuged at 8,000 G for 1 min; 500 ml of AW2 buffer was then added to the pellet, and the tube was centrifuged at 13,000 G for 3 min. The tube was removed and changed; then 50 µl of elution buffer (AE buffer) was added to the final DNA pellet. After incubation at 15-25°C for 1 min, the contents were centrifuged at 8,000 G for 1 min to collect the DNA [15,17].

### DNA amplification and sequencing

A 290-bp DNA fragment of *S. scabiei* *COX-1* was amplified by polymerase chain reaction (PCR) using the isolated genomic DNA as a template. The sequences of the forward and reverse primers were designed using the program Primer3Plus[18] based on the DNA sequence of an *S. scabiei* *COX-1* gene isolated from the Zi’gong rabbit (GenBank accession No. EU256389.1). The forward primer was 5’-TCTTAGGGGCTGGATTTAGTATG-3’ and the reverse primer was 5’-AGTTCCTCTACC AGTTCCAC-3’. PCR volumes were 20 μl, consisting of 10 μl Master Mix (Thermo Scientific), 1 μl each of forward and reverse primers, 6 μl distilled water, and 2 μl DNA template. PCR amplification was performed in an iCycles IQ (Biorad) with an initial denaturation at 95°C for 5 min, followed by 35 temperature cycles, each consisting of a 30 s denaturation at 95°C, a 60 s annealing at 50°C, and a 60 s extension at 72°C. After 35 cycles, there was a final extension for 5 min at 72°C. The amplified PCR product was electrophoresed on a 2% agarose gel in 1× Tris-borate-EDTA buffer with pH 8.3 and 0.03 μl ethidium bromide. The gel was visualized under ultraviolet light [15,19].

The PCR product was then purified according to the protocol of theBigDye XTerminator™ Purification Kit (Thermo Scientific, USA) and double-sequenced with the PCR forward and reverse primers using an ABI PRISM 310 Genetic Analyzer (Applied Biosystems).

### Bioinformatics analysis

To confirm the sequencing output, the sequence resulting from the reverse primer was inverted and aligned to that from the forward primer using the program Clone Manager Professional 9 Version 9 for Windows (Scientific & Educational Software; http://www.scied.com) using the DNA sequence with GenBank accession No. EU256389.1 mentioned above as a reference. The confirmed *COX-1* sequences of the Mojokerto rabbits and Lamongan goats were deposited in GenBank (https://www.ncbi.nlm.nih.gov/genbank/) under accession codes MH077557 (Lamongan goat) and MH077558 Mojokerto rabbit.

For comparison with other related DNA sequences, the *COX-1* DNA fragment sequence from the Mojokerto rabbit (GenBank accession: MH077558) was input as a query search with the program nucleotideBLAST. All hits with a nucleotide identity of 80% or higher, along with all sequences resulting from this investigation, were aligned using the ClustalW2 program [20]. This alignment was subsequently used to build a phylogenetic tree with the Neighbor-Joining method, in theMEGA6 program (https://www.megasoftware.net/) [21,22], with a *COX-1* sequence from *Megaselia* spp. (GenBank accession No. KT103510.1) as an outer group.

## Results

**Figure-1:**
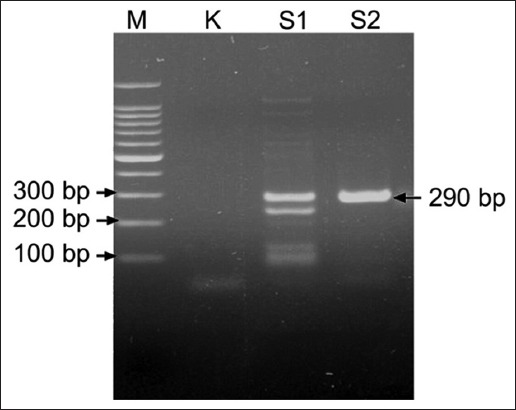
Polymerase chain reaction products of *Sarcoptes scabiei* from goat-Lamongan (S1) and rabbit-Mojokerto (S2), negative control (K), marker (M).

*S. scabiei* mites were isolated from Lamongan goats and Mojokerto rabbits, with clinical symptoms: Thickening of the skin, crust formation, and hair loss on the areas around the eyes, ears, mouths, and legs. The results of 2% agarose gel electrophoresis of *COX-1* DNA from *S. scabiei*, amplified with the primer pairs described above, show the PCR products were around 290bp long, indicating bands at positions between 200 bp and 300 bp markers (Figure-1).

**Figure-2:**
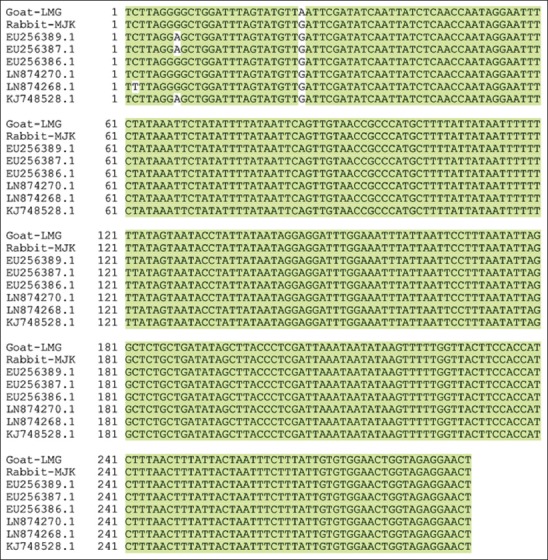
Sequence alignment of the polymerase chain reaction products of the cytochrome c oxidase-1 gene of *Sarcoptes scabiei* from Lamongan goats and Mojokerto rabbits with data from GenBank.

Sequence analyses confirmed that the PCR products were a partial CoDing Sequence (CDS) of the *COX-1* gene (Figure-2). Sequence comparison showed that the partial CDS of the *S. scabiei* *COX-1* gene isolated from Lamongan goats is homologous to that from Mojokerto rabbits, with an identity of 99%. Out of 290 nucleotides aligned, only one nucleotide was different. An adenine was found in the CDS of the *S. scabiei* *COX-1* gene isolated from Lamongan goats, in place of guanine in that position in the sequence from Mojokerto rabbits. The guanine residue is conserved in other *S. scabiei* *COX-1* gene. A BLASTN analysis against the National Center for Biotechnology Information (NCBI) nucleotide collection (nr) database reveals about 19 *S. scabiei* isolates from animal species from various countries, which were similar identical at nucleotide lengths of 290 bp.

**Figure-3:**
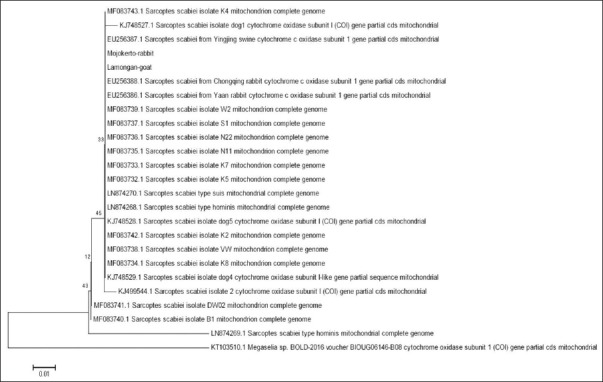
Phylogenetic analysis of partial CDS of the cytochrome c oxidase subunit 1 gene of *Sarcoptes scabiei*, isolated from several different species. The genes from Lamongan goats and Mojokerto rabbits are introduced in this article, while the other sequences were obtained from the National Center for Biotechnology Information (NCBI) nucleotide database, their accession numbers are shown. All sequences were aligned using ClustalW2, and the cladogram was built using the Neighbor-Joining method [20]. Bootstrap test values, which were resulted from 500 replications are indicated for each branch. The sequences with the NCBI accession number KT103510.1 are from *Megaselia* spp. BOLD-2016 voucher BIOUG06146-B08 and were used as an outlier in this analysis.

The phylogenetic tree of the *COX-1* gene of *S. scabiei* mites from Lamongan goats and Mojokerto rabbits was constructed using the MEGA6 program’s Neighbor-Joining method. This tree shows that the gene is relatively close to 19 *S. scabiei* isolates obtained from the NCBI nucleotide database with their accession numbers [20] (Figure-3). Phylogenetic analysis of the *COX-1* nucleotide sequences revealed three clades of *S. scabiei*: (1) A large, unresolved clade, including sequences from Lamongan goats and Mojokerto rabbits, and sequences obtained from the GenBank of *S. scabiei* from various hosts and regions; (2) a clade of *S. scabiei* from Australian wombats and *S. scabiei* isolate B1; and (3) a clade of *S. scabiei* type *hominis* from Australia and *Megaselia* spp. from Canadian insects, used as an outer group in this analysis (Figure-3).

## Discussion

The sequence alignment has shown that the partial CDS of *S. scabiei* *COX-1* gene isolated from Lamongan goats is almost identical to that from Mojokerto rabbits. Out of 290 nucleotides aligned, only one nucleotide was different, an adenine was found in the CDS of the *S. scabiei* *COX-1* gene isolated from Lamongan goats in place of guanine in that sequence from Mojokerto rabbits. The guanine residue is conserved in other *S. scabiei* *COX-1* genes. The *COX-1* gene has conserved region and variable region, and differences are possible in the variable region. However, this mutation is most likely silent [9,16].

Based on the alignment results, the *COX-1* mitochondrial *S. scabiei* gene has a high level of identity among isolates. Possibly, the *S. scabiei* infecting Lamongan goats was the same as the one infecting Mojokerto rabbits. This observation indicates that *S. scabiei* isolates from Lamongan goats and Mojokerto rabbits are the same species, and the *COX-1* mitochondrial *S. scabiei* did not evolve. Makouloutou *et al*. [9] have stated that the *S. scabiei* causes scabies in most Japanese wild animals, similar to that in wild animals on other continents.

Research into *S. scabiei* mites’ genetics is still underway. Some research based on studies using the second internal transcribed spacer (ITS2) of the ribosomal RNA gene. The ITS2 gene has a conserved region and a variable region, at the genus level, it has the same conserved region, but the variable region will be different depending on the species. Taxonomically, *S. scabiei* has different varieties based on the host’s origin and shows cross-infectivity to a low degree between hosts, because each mammal has a specific receptor. *S. scabiei* morphology from various mammalian hosts and various regions indicates that it is a single species, but with heterogeneous variable region [4,13,23,24]. Erster *et al*. [25] reported that an analysis of mitochondrial *COX-1* did not show a correlation between host preference and genetic identity in *S. scabiei* from pet, farm, and wild hosts in Israel.

The phylogenetic analysis (Figure-3) shows relatively low bootstrap values, indicating the *COX-1* fragment sequences analyzed are rather difficult to resolve. Indeed, the sequences are highly similar. The sequences’ 290bp length may also contribute to these low bootstrap values. However, from the phylogenetic analysis, it is clear that *S. scabiei* from Lamongan goats and Mojokerto rabbits is relatively close to *S. scabiei* from various hosts such as swine, dog, rabbit, and Australian and Chinese marsupials. However, *Megaselia* spp. from Canadian insects is an outer group. Scabies is highly infectious and transmitted through direct contact, so it is possible to transmit it between goats kept in one cage, as well as between rabbits that are kept in one cage with poor sanitation. According to Arlian *et al*. [10], *S. scabiei* cross-infestation between different hosts does not occur in wild animals. In our study, *S. scabiei* mites were isolated from goat farms in the Lamongan area, where each farmer had around five to six animals in one cage, and they showed clinical symptoms of scabies. Similarly, *S. scabiei* isolated from rabbits showing clinical symptoms of scabies came from rabbit farms in Mojokerto, which 6-10 rabbits are kept in one cage with poor sanitation, so transmission between rabbits is easy. Transmission within a single species is possible because individuals have the same receptor. *S. scabiei* mites have adapted well to their hosts. Adaptation to the host and geographical segregation will reduce transmission between *S. scabiei* species. Understanding the transmission of *S. scabiei* has important implications in epidemiological studies for developing scabies control strategies in animals and humans [24,26,27].

## Conclusion

The PCR products of *S. scabiei* *COX-1* gene isolated from Lamongan goats and Mojokerto rabbits were around 290 bp in length. Sequences showed a similarity of more than 99%. The sequences of the *COX-1* gen *S. scabiei* from Lamongan goats and Mojokerto rabbits were relatively close to those of the *COX-1* gene of *S. scabiei* obtained from the NCBI databases from various hosts.

## Authors’ Contributions

NDRL, AR, and DH planned and designed the study. DC and KD collected samples and carried out the work. NDRL and DH helped with PCR examination and manuscript writing. AR performed the bioinformatics data analysis. All authors read and approved the final manuscript.
